# Draft Genome Sequences of *Lactobacillus equicursoris* CIP 110162^T^ and *Lactobacillus* sp. Strain CRBIP 24.137, Isolated from Thoroughbred Racehorse Feces and Human Urine, Respectively

**DOI:** 10.1128/genomeA.00663-13

**Published:** 2013-08-22

**Authors:** Sylvie Cousin, Valentin Loux, Laurence Ma, Sophie Creno, Dominique Clermont, Chantal Bizet, Christiane Bouchier

**Affiliations:** Institut Pasteur, Centre de Ressources Biologiques de l’Institut Pasteur, Département Microbiologie, Paris, Francea; Institut Pasteur, Plate-forme Génomique, Département Génomes et Génétique, Paris, Franceb; INRA, UR1077 Mathématique, Informatique et Génome, Jouy-en-Josas, Francec

## Abstract

We report the draft genome sequences of strain *Lactobacillus equicursoris* CIP 110162^T^, isolated from racehorse breed feces, and *Lactobacillus* sp. strain CRBIP 24.137, isolated from human urine; the two strains are closely related. The total lengths of the 116 and 62 scaffolds are about 2.157 and 2.358 Mb, with G+C contents of 46 and 45% and 2,279 and 2,342 coding sequences (CDSs), respectively.

## GENOME ANNOUNCEMENT

*Lactobacillus equicursoris* strain CIP 110162^T^ was isolated from the feces of a healthy thoroughbred racehorse in Japan, whereas *L. equicursoris* strain 66C is a clinical isolate from a human urine sample; 66C was deposited at the collection of the Institut Pasteur under the name CRBIP 24.137. It was previously demonstrated that strain CIP 110162^T^ formed a subcluster in the *Lactobacillus delbrueckii* phylogenetic group and was closely related to *L. delbrueckii* but yet genetically distinct from its phylogenetic relatives (1). Sequence analysis of the 16S rRNA gene revealed that strain CRBIP 24.137 shows 99.1% similarity with the strain *L. equicursoris* CIP 110162^T^. DNA–DNA relatedness between strain 66C and the closely related type strains was more than the 70% cutoff value that is recommended for species delineation (2). The average nucleotide identity between the 2 genomes is 99.8% (3). These figures confirm that the two strains belong to the same species.

Here, we report the genome sequences of *L. equicursoris* CIP 110162^T^ and *Lactobacillus* sp. strain CRBIP 24.137, obtained using a whole-genome strategy based on Illumina paired-end sequencing, with insert lengths of about 420 and 352 bp, respectively (Illumina HiSeq 2000), as observed on Agilent high-sensitivity DNA kit. Quality-filtered reads (71,743,098 and 68,403,694 reads, 97 and 97.7 bases mean read length, and ~3,040 and 2,790-fold coverage, respectively) were assembled using ABySS software (version 1.2.6 [4]) with different *k*-mer lengths (*k*) of 25, 30, 40, and 60. Scaffolds with maximum lengths of 122,530 and 254,168 bases were obtained with *k* values of 40 and 60, respectively.

The draft genomes for CIP 110162^T^ and CRBIP 24.137 consist of 2,156,576 and 2,357,741 nucleotides (nt) split into 116 and 62 scaffolds and with G+C contents of 46 and 45%, respectively. The scaffolds were annotated with the AGMIAL platform (5), an integrated bacterial genome annotation system. The prediction of coding sequences used the self-training gene detection software SHOW based on hidden Markov models (http://genome.jouy.inra.fr/ssb/SHOW/). tRNAs and rRNAs were detected using tRNAscan-SE (6) and RNAmmer (7) softwares, respectively. The numbers of predicted coding sequences (CDSs) were 1,937 and 2,104, respectively; 3 rRNA operons with 1 copy each of 23S, 5S, and 16S and 55 tRNA genes were detected for strain CIP 110162^T^, while 5 rRNA operons with 2 and 3 copies of 5S and 16S, respectively, and 72 tRNA genes were detected for strain CRBIP 24.137.

### Nucleotide sequence accession numbers. 

The strains are publicly available in two European collections under no. CIP 110162^T^, DSM 19284^T^, CRBIP 24.137, and DSM 23909. The draft of the whole-genome sequencing project has been deposited in EMBL under the accession no. CAMA01000001 to CAMA01000232 and CALZ01000001 to CALZ01000161, respectively. The versions described in this paper are the first versions.
